# Reproducibility of range of motion and muscle strength measurements in patients with hip osteoarthritis – an inter-rater study

**DOI:** 10.1186/1471-2474-13-242

**Published:** 2012-12-06

**Authors:** Erik Poulsen, Henrik Wulff Christensen, Jeannette Østergaard Penny, Søren Overgaard, Werner Vach, Jan Hartvigsen

**Affiliations:** 1 Institute of Sports Science and Clinical Biomechanics, University of Southern Denmark, Odense, Denmark; 2 Nordic Institute of Chiropractic and Clinical Biomechanics, Odense, Denmark; 3 Department of Orthopaedic Surgery and Traumatology, Odense University Hospital, Odense, Denmark; 4 Institute of Clinical Research, University of Southern Denmark, Odense, Denmark; 5 Institute of Medical Biometry and Medical Informatics, University of Freiburg, Freiburg, Germany

**Keywords:** Hip, Examination, Inter-observer, Reliability, Osteoarthritis, Hip

## Abstract

**Background:**

Assessment of range of motion (ROM) and muscle strength is fundamental in the clinical diagnosis of hip osteoarthritis (OA) but reproducibility of these measurements has mostly involved clinicians from secondary care and has rarely reported agreement parameters. Therefore, the primary objective of the study was to determine the inter-rater reproducibility of ROM and muscle strength measurements. Furthermore, the reliability of the overall assessment of clinical hip OA was evaluated. Reporting is in accordance with proposed guidelines for the reporting of reliability and agreement studies (GRRAS).

**Methods:**

In a university hospital, four blinded raters independently examined patients with unilateral hip OA; two hospital orthopaedists independently examined 48 (24 men) patients and two primary care chiropractors examined 61 patients (29 men). ROM was measured in degrees (deg.) with a standard two-arm goniometer and muscle strength in Newton (N) using a hand-held dynamometer. Reproducibility is reported as agreement and reliability between paired raters of the same profession. Agreement is reported as limits of agreement (LoA) and reliability is reported with intraclass correlation coefficients (ICC). Reliability of the overall assessment of clinical OA is reported as weighted kappa.

**Results:**

Between orthopaedists, agreement for ROM ranged from LoA [-28–12 deg.] for internal rotation to [-8–13 deg.] for extension. ICC ranged between 0.53 and 0.73, highest for flexion. For muscle strength between orthopaedists, LoA ranged from [-65–47N] for external rotation to [-10 –59N] for flexion. ICC ranged between 0.52 and 0.85, highest for abduction. Between chiropractors, agreement for ROM ranged from LoA [-25–30 deg.] for internal rotation to [-13–21 deg.] for flexion. ICC ranged between 0.14 and 0.79, highest for flexion. For muscle strength between chiropractors, LoA ranged between [-80–20N] for external rotation to [-146–55N] for abduction. ICC ranged between 0.38 and 0.81, highest for flexion. Weighted kappa for the overall assessment of clinical hip OA was 0.52 between orthopaedists and 0.65 between chiropractors.

**Conclusions:**

Reproducibility of goniometric and dynamometric measurements of ROM and muscle strength in patients with hip OA is poor between experienced orthopaedists and between experienced chiropractors. Orthopaedists and chiropractors can to a moderate degree differentiate between hips with or without osteoarthritis.

## Background

In primary care, when patients over 40 years of age present with hip pain, the most common diagnosis is osteoarthritis (OA) [1,2]. A combination of radiographic signs and clinical findings is usually recommended for confirming the diagnosis. But although approximately half demonstrate definite radiological signs of OA [1], radiographs are not recommended solely for just confirming the diagnosis. Thus, the clinical exam is of key importance [3]. Clinical practice guidelines recommend assessment of range of motion (ROM) and muscle strength when adult patients present with hip pain [4] and the two clinical signs documented to correlate with hip OA besides pain are reduced ROM [5-8] and muscle strength [5,8-11]. Reduced ROM is further documented as a clinical predictor for hip OA [2,12] and in patients with mild symptomatic hip OA, specific ranges of reduced ROM are correlated with radiographic signs [13].

A number of studies have evaluated the reliability of ROM and muscle strength measurements in patients with hip OA and reported moderate to excellent reliability [6,7,14-19]. But the presence of methodological issues raises questions about the external validity of these results. Equipment ill-suited for clinical practice has been used [7,18] or the number of study subjects has been small, limiting the between-subject variation [6,14,16,17]. Inappropriate correlation coefficients have been reported [14,15] or reliability coefficients have been reported alone, ignoring agreement parameters [15,17,19]. Reliability coefficients indicate the procedure’s ability to discriminate between patients, whereas agreement parameters reflect error between repeated measurements [16,17]. So, when measurements are used to assess change over time, agreement parameters should be reported [20].

Intra-rater reproducibility is commonly found to be more reliable than inter-rater reproducibility because between-rater variability is eliminated [21-23]. In clinical or research settings, intra-rater reproducibility could be adequate where only one rater performs the measurements, whereas inter-rater reproducibility is essential for clinicians when follow-up consultations on the same patient are performed by different clinicians or when clinicians have to agree on a diagnosis. Three studies have examined inter-rater reliability of ROM measurements on hip OA patients but none reported agreement parameters [16,17,24]. One study reported inter-rater reliability on muscle strength measurements in hip OA patients but agreement parameters were not reported [17]. Only one study evaluating reproducibility among primary care clinicians has been identified [16].

Therefore, the primary purpose of this study was to assess the inter-rater reproducibility of passive ROM and muscle strength measurements in patients with unilateral hip OA among clinicians in both primary care and hospital secondary care. The secondary purpose was to assess the inter-rater reliability of the degree of clinical hip OA among the same clinicians based on findings of ROM and strength measurements.

## Methods

### Participants

**Figure 1:**
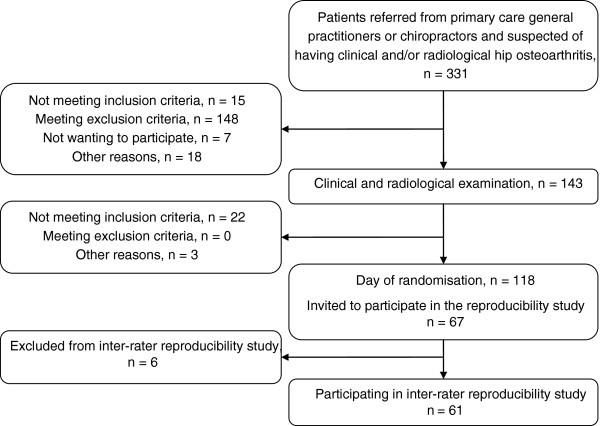
Flow chart of participants included in the study.

The study participants took part in a randomised clinical trial described elsewhere [25]. Recruitment of the participants is illustrated in Figure 1. Inclusion criteria included unilateral hip pain >3 months and unilateral radiographic hip OA on the painful side. The complete lists of inclusion and exclusion criteria are presented in Table 1. Prior to examination, each participant completed a questionnaire with details on age, gender, height, weight, side of hip pain, duration of complaint and pain severity. The participant reported average pain experienced during the previous week and worst pain experienced during the previous week.

**Table 1:** Inclusion and exclusion criteria for participants

**Inclusion criteria**	**Exclusion criteria**
− 40-80 years of age	− Bilateral hip pain
− Unilateral hip pain >3 months	− Previous hip or knee joint replacement surgery
− Radiographic hip osteoarthritis (OA) defined by joint space width measurements <2.00 mm or a side difference >10%	− Indication for hip joint replacement surgery within the next 6 months
− Ability to speak and read Danish	− Hip OA due to hip fracture or infection
	− Rating of worst hip pain during the last week as ≤2 on an 11-box numerical rating scale
	− Manual therapy for the hip within the last 12 months
	− Hip dysplasia; Center Edge angle <25 degrees and Acetabular index Angle >10 degrees
	− Local knee pain originating from the knee on the same side as the hip OA
	− Low back pain dominating over hip symptoms
	− Inflammatory joint disease
	− Polyarthritis defined as >3 regional sites
	− Cerebrovascular disease
	− Polyneuropathy or neuromuscular disease
	− Malignant disease
	− Conditions other than hip OA appearing to be the cause of the patient’s symptoms
	− Refusal to participate

Prior to their involvement, each participant received verbal and written information about the study and signed a written consent form. The study was granted approval by the Regional Ethics Committee of Southern Denmark, approval number S-20080027 and was registered and approved by the Danish Data Protection Agency, J.nr. 2008-41-1910.

### Raters

Four raters participated. There were two medical doctors from hospital care: one male senior orthopaedic surgeon specialising in hip surgery with clinical experience of >20 years and one female first year resident in orthopaedic surgery with 4 years’ experience. And there were two male chiropractors working in primary care, both with clinical experience of >20 years: one with 8 years of clinical interest in specific hip conditions and one with no specific interest or clinical experience with hip conditions. At the time of examination, these raters were aware of the inclusion- and exclusion criteria but had no prior knowledge of which side of the body involved the hip condition and they were blind to the radiographic findings.

### Setting and equipment

All examinations took place at Odense University Hospital, Denmark. Passive hip ROM was measured using a standard two-arm plastic goniometer, 30 cm, 0-360 degrees (deg.) with single deg. increments (MSD Europe bvba). Recordings were made to the nearest five deg. Hip muscle strength was measured in Newtons (N) using a hand-held dynamometer (HHD), model MicroFet II (Hoggan Health Industries Inc.). The goniometer and HHD were chosen as they are inexpensive and easy to implement in both primary and outpatient hospital care. It was decided to test them on raters with minimal protocol standardisation and without rigorous training.

### Procedures

The protocol for the examination procedures is attached as an appendix [see Additional file 1]. The aim of the protocol was to resemble test procedures used in daily practice and it was created by consensus between the raters.

A day was scheduled to familiarise raters with the use of the equipment and rehearse individual examination procedures. Two university students acted as study subjects. Initially, measurements for ROM and strength were included for all six directions of movement, i.e. extension, flexion, abduction, adduction, internal and external rotation. Strength testing in adduction was excluded due to consensus on issues concerning practicality and interpretation when examining this patient group. The procedure requires stability of the pelvis and opposite leg during testing and HHD placement includes lower leg strength. In order to detect differences in maximum strength in patients with early to mild hip osteoarthritis, it was decided to use a break test and not an isometric test [26]. The protocol was revised and a training day was scheduled with eight patients with hip pain and radiographic hip OA. Following the training session, corrections were made regarding the positioning of participants. The final protocol was approved by all raters. Measurements were performed on both hips.

On the days of data collection, four separate cubicles were created by room dividers with identical examination tables. Four participants were asked to each enter a cubicle, undress to their underwear and wait for a rater. Each participant was then examined by the four raters in turn, randomly rearranging the sequence of raters after each examination to minimise any possible learning effect. Raters were free to determine which hip to examine first. Communication between rater and participants regarding examination procedures was allowed but information pertaining to the participant’s case history was not. No communication between raters was allowed in between sessions. An assistant was assigned to each rater to record the result of the examination findings on a standardised form and to assist holding the goniometer during ROM in extension. ROM was measured once and muscle strength measured twice.

Following completion of all measurements, each rater independently assessed each hip for the degree of clinical hip OA and assigned it to one of three categories: no hip OA, mild hip OA or severe hip OA. The decision of the category was based on the opinion of each rater.

For generalisability and to obtain a representative study sample it was decided to include a minimum of 60 participants.

### Statistical analysis

Double data entry was performed by a person not involved in the study. Descriptive statistics are presented for participant characteristics. For the continuous variables of hip ROM and muscle strength, means and standard deviations (SDs) for each rater are reported, and since we were interested in the reproducibility between raters of the same profession, i.e. orthopaedists and chiropractors, pair-wise mean differences and SDs between raters of the same profession are reported. The value reported for muscle strength is an average of two measurements. Bland and Altman plots were inspected visually for indication of heteroscedasticity. Measurement error is reported as standard error of the measurement (SEM_agreement_) described by de Vet et al. and is reported for the purpose of comparison with other studies [20]. SEM_agreement_ incorporates measurement error between raters and error from interaction between raters and participants.

Agreement between raters is reported as 95% limits of agreement (LoA) as described by Bland and Altman where the clinical interpretation is based on the 95% range [27]. So, if the systematic rater error between two raters is zero, half the range can be considered the smallest detectable change (within 95% confidence). Percent agreements between raters are reported for ROM as agreement within 10 deg. for flexion and 5 deg. for all other ROMs. Ten deg. for flexion was chosen since the range in flexion is considerably larger. Clinically acceptable percent agreement between clinicians was set *a priori* to 75%. Reliability is reported with the intraclass correlation coefficient (ICC_2.1_) including 95% confidence intervals and is reported within raters of the same profession. Interpretation of ICC is according to the classification: < 0.69, poor; 0.70-0.79, fair; 0.80-0.89, good; 0.90-1.00, excellent [28]. Acceptable reliability was set *a priori* at ≥0.70 [29]. ICC_2.1_ was used in order to generalise the to a wider population of raters [30]. The reliability of the overall assessment of clinical hip OA is reported with Cohen’s weighted kappa. The interpretation of Cohen’s weighted kappa is according to the classification by Landis and Koch [31]: <0.00, poor; 0.00-0.20, slight; 0.21-0.40, fair; 0.41-0.60, moderate; 0.61-0.80, substantial, 0.81-1.00, almost perfect. Kappa is weighted as 1.0 / 0.5 / 0.0. Acceptable kappa values were set *a priori* at ≥0.60. Analysis was performed using Stata 10 software (StataCorp, Texas, USA).

## Results

Sixty-seven participants were invited to take part in the study. Three were excluded due to bilateral hip pain, one due to neuropathy, one for having no radiographic signs of hip OA and one failed to attend, resulting in 61 participants. Inclusion of participants took place from January 2009 to September 2009 and a total of 5 days evenly distributed throughout the period were used for examinations. The senior orthopaedic surgeon was not available for one of these days, so a total of 48 participants were assessed for comparison between the two orthopaedists. Results are only presented for the hip with clinical and radiographic OA. Descriptive participant characteristics are listed in Table 2. Means and SDs for ROM and strength measurements for all four raters are listed in Table 3 as well as pair-wise mean differences and SDs between orthopaedists and between chiropractors. SEM_agreement_, percent agreement for ROM, LoA and ICC for the pair-wise comparison are also listed in Table 3.

**Table 2:** Characteristics of participants

	
Involved side, right / left (n)	36 / 25
Gender, men / women (n)	29 / 32
Mean age in years (SD)	65.6 (8.0)
Mean body mass index in kg/m^2^ (SD)	26.8 (3.4)
Mean duration of symptoms in months (SD)	37 (32)
Mean average level of pain during the last week* (SD)	4.7 (1.8)
Mean worst level of experienced pain during the last week* (SD)	5.7 (2.0)

**Table 3:** **Inter-rater reproducibility of hip range of motion (deg.) and muscle strength (N)** f**or 2 orthopaedists and 2 chiropractors**

**Hip range of motion measured with a goniometer to the nearest 5 deg.**	**Ortho 1 (deg.) Mean (SD)**	**Ortho 2 (deg.) Mean (SD)**	**Mean difference (SD)**	Percent agreement (%)*	**LOA (deg.)**	**SEM†**	**ICC (95% CI)**
Flexion (n = 48)	113 (18)	106 (14)	7 (10)‡	71	-12 – 26	8	0.73 (0.38 – 0.87)
Extension (n = 48)	6 (6)	4 (8)	2 (5)‡	79	-8 – 13	4	0.68 (0.46 – 0.81)
Abduction (n = 48)	29 (10)	26 (8)	3 (7)‡	67	-12 – 17	6	0.63 (0.41 – 0.76)
Adduction (n = 48)	17 (7)	17 (7)	0 (6)	77	-11 – 12	4	0.65 (0.45 – 0.79)
Internal rotation (n = 48)	4 (14)	12 (14)	-8 (10)‡	42	-28 – 12	9	0.63 (0.17 – 0.82)
External rotation (n = 48)	28 (11)	33 (7)	-4 (9)‡	69	-21 – 13	6	0.53 (0.26 – 0.72)
**Hip muscle strength measured with a dynamometer in Newton (N)**	**Strength (N)**	**Strength (N)**			**(N)**		
Abduction (n = 48)	213 (65)	212 (67)	0 (37)		-72 – 72	26	0.85 (0.74 – 0.91)
Flexion (n = 48)	167 (42)	185 (50)	-21 (41)‡		-101 – 59	32	0.55 (0.28 – 0.73)
Internal rotation (n = 48)	151 (38)	146 (36)	5 (36)		-66 – 76	26	0.52 (0.29 – 0.70)
External rotation (n = 48)	123 (34)	132 (39)	-9 (28)‡		-65 – 47	21	0.68 (0.49 – 0.81)
**Hip range of motion measured with a goniometer to the nearest 5 deg.**	**Chiro 1 (deg.) Mean (SD)**	**Chiro 2 (deg.)** **Mean (SD)**	**Mean difference (SD)**	Percent agreement (%)*	**LOA (deg.)**	**SEM†**	**ICC (95% CI)**
Flexion (n = 61)	112 (13)	108 (15)	4 (9)‡	83	-14 – 21	7	0.79 (0.63 – 0.88)
Extension (n = 61)	9 (5)	14 (5)	-6 (5)‡	66	-16 – 5	5	0.33 (0.06 – 0.61)
Abduction (n = 61)	29 (9)	22 (9)	7 (8)‡	46	-8 – 23	8	0.45 (0.01 – 0.71)
Adduction (n = 61)	17 (7)	14 (5)	3 (8)‡	61	-13 – 19	6	0.14 (0.09 – 0.36)
Internal rotation (n = 61)	13 (10)	11 (16)	2 (14)	31	-25 – 30	10	0.44 (0.21 – 0.62)
External rotation (n = 61)	32 (9)	30 (12)	2 (11)	59	-19 – 23	8	0.48 (0.27 – 0.65)
**Hip muscle strength measured with a dynamometer in Newton (N)**	**Strength (N)**	**Strength (N)**			**(N)**		
Abduction (n = 61)	141 (42)	186 (61)	-45 (51)‡		-146 – 55	48	0.38 (0.00 – 0.64)
Flexion (n = 61)	165 (50)	178 (65)	-11 (35)‡		-79 – 56	26	0.81 (0.69 – 0.88)
Internal rotation (n = 61)	142 (43)	190 (49)	-48 (34)‡		-115 – 19	42	0.48 (0.09 – 0.77)
External rotation (n = 61)	120 (34)	150 (48)	-30 (26)‡		-80 – 20	28	0.65 (0.00 – 0.82)

deg., degrees; N, newton; SD, standard deviation; LOA, limits of agreement; SEM, standard error of the measurement; ICC, intraclass correlation coeffficient; CI, confidence interval.

*Percent agreement. Difference ≤ 5^o^ between examiners except for flexion ≤10^o^.

†SEM_agreement_.

‡Statistically significant differences between means, p<0.05.

Statistically significant differences (p<0.05) were found in general between all pair-wise measurements. But specific patterns for ROM measurements were not noted for the pair-wise comparisons. One chiropractor demonstrated systematically higher values for all hip muscle strength measurements. The systematic difference for the individual measurements is further reflected in the LoA with the upper and lower limits deviating non-symmetrically from zero. Visual inspection of the Bland and Altman plots did not indicate heteroscedasticity.

**Figure 2:**
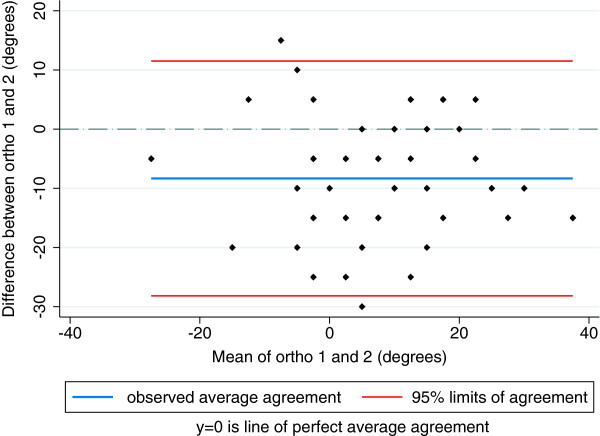
Limits of agreement between two orthopaedists for hip internal rotation range of motion (degrees).

**Figure 3:**
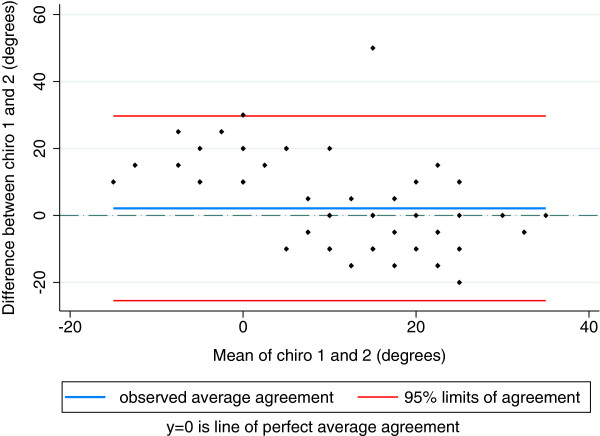
Limits of agreement between two chiropractors for hip internal rotation range of motion (degrees).

Percent agreement for ROM between orthopaedists ranged between 42 and 79%. Between chiropractors, the range was 31 – 83%. Between orthopaedists, LoA for ROM ranged from [-8-13 deg.] for extension to [-28-11 deg.] for internal rotation and between chiropractors the range was from [-13-21 deg.] for flexion to [-25-30 deg.] for internal rotation. LoA for internal rotation between orthopaedists are illustrated in Figure 2 and between chiropractors in Figure 3. Reliability for ROM between orthopaedists ranged from 0.53 (95% CI 0.26-0.72) for external rotation to 0.73 (0.38-0.87) for flexion. Between chiropractors, the range was 0.14 (-0.09-0.36) for internal rotation to 0.79 (0.63-0.88) for flexion.

**Figure 4:**
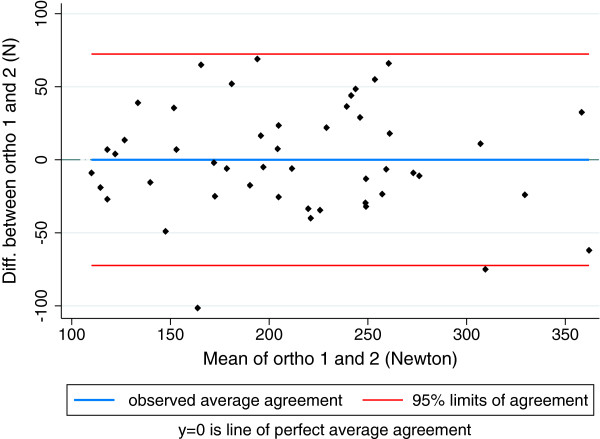
Limits of agreement between two orthopaedists for abduction hip strength (Newton).

**Figure 5:**
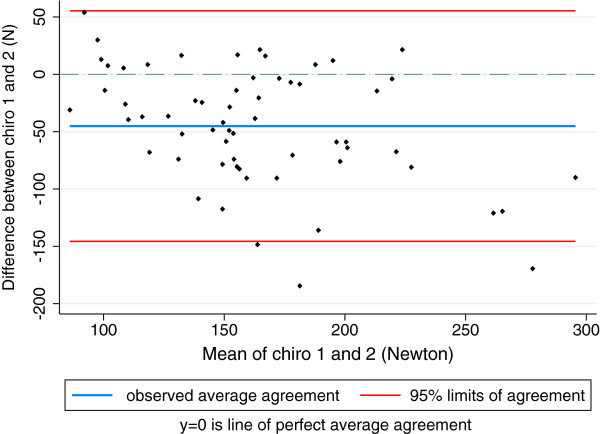
Limits of agreement between two chiropractors for abduction hip strength (Newton).

For muscle strength, LoA between orthopaedists ranged from [-65-47N] for external rotation to [-101-59N] for flexion and between chiropractors, the range was from [-80-20N] for external rotation to [-146-55N] for abduction. LoA for abduction between orthopaedists are illustrated in Figure 4 and between chiropractors in Figure 5. ICC for orthopaedists ranged from 0.52 (0.29-0.70) for internal rotation to 0.85 (0.29-0.70) for abduction. For chiropractors, the ICC ranged from 0.38 (0.00-0.64) for abduction to 0.81 (0.69-0.88) for flexion.

Between orthopaedists, reliability (weighted kappa) for the degree of clinical hip OA based on ROM and muscle strength assessment was 0.52 and between chiropractors, 0.65.

## Discussion

To our knowledge, this is the first study involving clinicians from both primary care (chiropractors) and hospital secondary care (orthopaedists). We found generally poor to moderate inter-rater reproducibility for all ROM and muscle strength measurements both between orthopaedists and between chiropractors. Acceptable reproducibility was found only for hip ROM in flexion, both between orthopaedists and between chiropractors. Reliability for the assessment of clinical hip OA is moderate both between orthopaedists and between chiropractors.

### Clinical interpretation

When incorporating the measurement error into a clinical context, the wide limits of all LoAs for ROM for both orthopaedists and chiropractors indicate that an effect following intervention should be a minimum of 17 deg. for flexion, 10 for extension, 15 for abduction, 12 for adduction and 20 for internal rotation and 17 for external rotation before it with (95% confidence) can be distinguished from random fluctuations due to measurement error, if measured by two different raters. Considering the normal range for flexion and abduction, this is possible but unlikely for extension, adduction and internal and external rotation. Interpretation of the results for flexion and abduction though must be done with care as Müller and Büttner argue the ICC is “dependent on the range of the measuring scale” [32]. So the larger the scale, the higher the coefficient and the range for flexion and abduction is considerably larger than the other ROMs of the hip. The clinical interpretation of reliability must involve the lower 95% CIs which further reflect the poor to moderate findings [33]. Only muscle strength for abduction between orthopaedists demonstrated acceptable lower 95% CI of 0.74 and between chiropractors for flexion with lower 95% CI of 0.69.

For hip muscle strength, the same interpretation of LoA is not possible as muscle strength diminishes with each decade and is up to 50% higher in males [26]. Further, variation in force applied between raters can be significantly different and between raters of opposite sex [34,35]. The latter was not apparent between the orthopaedists as mean flexion and external rotation was significantly higher for the female orthopaedist.

Observing the results between the two orthopaedists and the two chiropractors did not give any indication of one group of professionals producing more reliable measurements than the other. However, the reliability measures between chiropractors were lower when assessing both ROM and muscle strength and could reflect that their clinical practice clientele are typically not solely hip pain patients. The variation between the orthopaedic surgeon and the first year intern probably reflects the difference in experience.

The level of standardisation and minimal training is likely to have influenced the systematic differences seen in almost all individual measurements. As differences were not systematically higher for one specific rater across individual ROMs, individual habits such as placement of the instrument and rater’s force are likely to be the cause. The poor results of ROM in internal and external rotation could reflect participants being positioned supine and not sitting, as position is known to influence the precision of individual measurements [17]. One chiropractor had higher measurements for all strength tests, which is likely to be attributed to the force generated during the break test and in inter-rater variability interpreting when the break test is accomplished. The recorded variation in muscle strength could be due to fatigue from repetitive testing as participants were examined four times. We consider this effect minimal, as examinations were scheduled with a 15-minute interval and each session of strength testing lasted no more than 5 minutes. This allowed time for the ROM examination, a resting period for the participant and a change-over of raters. The results are also likely to be influenced by the orthopaedists or chiropractors having limited experience with the HHD. The procedures were tested in a validation study as part of the randomised clinical trial mentioned earlier (data not published). The rater tested had similar experience with the HHD and demonstrated similar levels of intra-rater reliability but with much narrower LoA intervals. For ROM measurements, the rater demonstrated clinically acceptable intra-rater reproducibility without routine use of a goniometer in practice.

### Comparison with other studies

Several studies have documented from poor to excellent inter-rater reliability of ROM in patients with hip OA using a goniometer. Sutlive et al. found fair to good reliability but agreement parameters were not reported [19]. Holm et al. studied teams of raters but results for mean measurements of each ROM were combined from all raters [14]. Cibere et al. found clinically acceptable reliability both before and after standardisation of ROM and muscle strength measurements but they did not incorporate variance components from the patients or random error and agreement parameters were not reported [17]. Theiler et al. reported reliability coefficients similar to those in our study but used Pearson’s correlation coefficient which does not incorporate systematic differences between raters [15]. For hip muscle strength, Arnold et al. found excellent inter-rater reliability using a different HHD model but subjects were a mix of patients with both hip and knee OA [36]. Studies have documented good to excellent intra- and inter-rater reliability on healthy subjects using goniometer and HHD but they are not comparable to hip OA patients as age and disease characteristics influence the variation between subjects [21-23,35,37,38].

### Study limitations

There are a number of limitations associated with this study. First, raters were aware of the participant’ inclusion criterion of unilateral clinical and radiographic hip OA, so in the context of the clinical setting, no other hip conditions had to be considered. Second, the study did not involve rigorous training of the raters; however, we were interested in results reflecting current clinical practice. Several studies have reported on the added effect of protocol standardisation and rigorous training in musculoskeletal medicine [17,39,40] and such training could potentially result in better agreement. Third, the raters had prior knowledge of patients having unilateral clinical and radiographic hip OA which could inflate reliability coefficients. When one hip was examined, the rater would know if the other hip would be affected by OA or not. Fourth, the orthopaedic surgeon was not available for one of the examination sessions, so only 48 participants were included in the analysis between orthopaedists, instead of the 61 originally recruited. Fifth, the assessment of clinical hip OA was based solely on ROM and muscle strength evaluation. In clinical practice, a more extensive list of individual tests is used as well as information from the patient’s case history. It is further possible that the overall assessment was influenced by indications of a procedure being painful, to which the raters were not blinded. Sixth, we decided to omit adductor strength testing even though adductor strength has been documented to be reduced in patients with hip OA [5,9]. But measurement equipment has not been suitable for the clinical setting and in this patient group we concluded on the training day that stability of the pelvis and opposite leg were insufficient. We are aware that reproducibility of adductor strength testing by HHD on young healthy subjects has been reported as clinically acceptable [41]. Last, differentiation between levels of clinical hip OA following the overall assessment was only made from mild to severe hip OA. In the assessment of radiographic hip OA, it is common to categorise into none, mild, moderate and severe.

The literature on reproducibility of the clinical hip examination in patients with hip OA is limited and heterogeneous but recently the first set of guidelines on the reporting of reliability and agreement studies was published [33]. As patient characteristics differ in symptom and disease severity in primary and hospital care, future studies should take place in the setting where patient populations are examined and managed and involve clinicians from the same setting. To improve external validity, more than two clinicians should be included and selected randomly from an appropriate population of clinicians.

## Conclusions

When using goniometry for the assessment of hip range of motion and hand-held dynamometry for hip muscle strength in patients with hip osteoarthritis, reproducibility of individual measurements was generally poor between a pair of orthopaedists and a pair of chiropractors, indicating standardisation and rigorous training would be essential if this were to be improved. Both orthopaedists and chiropractors have a moderate ability to differentiate between hips without clinical osteoarthritis and hips assessed as having either mild or severe clinical osteoarthritis.

## Abbreviations

OA: Osteoarthritis; ROM: Range of motion; Deg: Degrees; HHD: Hand-held dynamometer; N: Newton; ICC: Intraclass correlation coefficient; SD: Standard deviation; CI: Confidence interval; LoA: Limits of agreement.

## Competing interests

All authors declare that they have no competing interests.

## Authors’ contributions

EP, HWC, SO and JH contributed to the conception and design of the study. EP, HWC, JØP and SO participated in the data collection. EP drafted the manuscript and performed the statistical analysis. EP, HWC, SO, WV and JH participated in the interpretation of the data. All authors participated in the critical revision of the article and made important contributions to the content. All authors read and approved the final manuscript.

## Pre-publication history

The pre-publication history for this paper can be accessed here:

http://www.biomedcentral.com/1471-2474/13/242/prepub

## Supplementary Material

Additional file 1 Examination protocol. Click here for file
