# Supplementary material for: Reproducibility of range of motion and muscle strength measurements in patients with hip osteoarthritis – an inter-rater study
Source: BMC Musculoskelet Disord. 2012 Dec 6;13:242. doi: 10.1186/1471-2474-13-242 (PMC3565957; doi:10.1186/1471-2474-13-242)
Supplement: Additional file 1 — Examination protocol. [file 1471-2474-13-242-S1.pdf]

APPENDIX 1

Protocol for examination of passive hip range of motion

Hip flexion – supine

The participant is supine with arms by their sides (alternatively, crossed across the chest if the rater considers the arms interfere with placement of the goniometer). The rater is standing on the same side as the examined hip. The rater flexes the examined hip to the point of maximal passive flexion, either by observing the raising of the opposite thigh or by felt restriction of the thigh. The goniometer is centred at the greater trochanter with the stabilising arm horizontal and parallel to the trunk of the patient and the moveable arm parallel to the femur. The degree of flexion is conveyed to the assistant.

Hip abduction - supine

The participant is supine. The rater is standing on the same side as the examined hip. The goniometer is centred at a point midway between the anterior superior iliac spine (ASIS) and the symphysis pubis. The stabilising arm is parallel to the midline of the participant and the participant is asked to hold the stabilising arm against his/her lower abdomen with instruction not to move it. The moving arm is placed parallel to the thigh. With one hand, the rater stabilises the pelvis by holding one hand around the opposite ASIS. With the other hand, the examined leg is moved into abduction until resistance is felt. Attention is paid to not externally rotate the hip when it is moved into abduction. The movable arm on the goniometer is realigned with the examined thigh and the degree of abduction is conveyed to the assistant.

Hip adduction - supine

The participant, rater and placement of goniometer are positioned as in hip abduction. The rater moves the examined leg into adduction across the non-examined leg until resistance is felt. Attention is paid to not externally rotate the hip when it is moved into adduction and attempt is made to avoid the non-involved thigh resisting the movement of the examined leg. The movable arm on the goniometer is realigned with the examined thigh and the degrees of adduction are conveyed to the assistant.

Hip internal rotation - supine

The participant is supine. The rater is standing on the same side as the examined hip. The rater flexes the examined leg to 90 degrees hip and knee flexion. The goniometer is placed with the centre at the tip of the patella and both arms facing distally along the shaft of the tibia. The longitudial plane is defined to be that parallel to the body. The rater moves the hip into full internal rotation until resistance is felt. The moveable arm of the goniometer is realigned with the starting position of the tibia and the degrees of internal rotation are conveyed to the assistant.

Hip external rotation - supine

The participant, rater and placement of goniometer are positionen as in internal rotation. The rater moves the hip into full external rotation until resistance is felt. The degrees of external rotation are conveyed to the assistant.

Hip extension - prone

The participant is prone. The rater is standing on the opposite side to the examined hip. The rater stabilises the pelvis on the side of examination by placing one hand distal to the iliac crest with the heel of the hand on the posterior superior iliac spine. The assistant is asked to place the goniometer on the side of the thigh with the centre at the trochanter major and the arms parallel to the thigh. The rater lifts the thigh into extension until resistance is felt. The assistant repositions the movable arm so it is parallel to the thigh and records the degrees of extension.

Protocol for examination of hip muscle strength

During all procedures of muscle testing a break test is applied. The participant is asked to increase the resistance of the force applied as the rater increases the opposite force until resistance of the force by the participant is "broken".

<u>Hip abduction – side lying</u>

The participant is lying on their side with the examined side up. The opposite leg is flexed 45 degrees at the hip and knee for stability. The rater stands behind the participant with one hand stabilising the pelvis. The participant is asked to lift the leg 5 cm into abduction with 5 degrees of hip extension in order to avoid the use of hip flexors during abduction. The rater places the dynamometer 10 cm proximal to the knee joint. The participant is instructed to hold the leg in the placed position while the rater gradually increases the opposite force until the leg "gives".
The recorded degree of maximal force is conveyed to the assistant.

<u>Hip flexion - sitting</u>

The participant is seated on the examination table with their legs hanging over the side of the table avoiding the back of knees making contact with the table. In order to minimise co-contraction of the arm and torso musculature during test procedure, the arms of the participants are placed behind their back and the participant is asked to avoid co-contraction. The rater is standing beside the hip being tested. The participant is asked to lift the examined leg 10 degrees from the table and the dynamometer is placed 10 cm proximal to the patella. The participant is instructed to hold the leg in the placed position while the rater gradually increases the opposite force until the thigh "gives".

The recorded degree of maximal force is conveyed to the assistant.

Hip internal rotation - sitting

The participant placement and instruction are the same as that for flexion. The rater is kneeling in front of the participant next to the leg being tested. One hand is stabilising the knee to minimise lateral knee movement. The dynamometer is placed on the lateral side of the lower leg approximately 5 cm proximal to the lateral malleoli. The participant is instructed to hold the leg in the placed position while the rater gradually increases the force into external rotation until the lower leg "gives".

The recorded degree of maximal force is conveyed to the assistant.

Hip external rotation - sitting

The participant placement and instruction are the same as for flexion. The rater is kneeling in front of the participant next to the leg opposite the one being tested. One hand is stabilising the knee to minimise lateral knee movement. The dynamometer is placed on the medial side of the lower leg

approximately 5 cm proximal to the medial malleoli. The participant is instructed to hold the leg in the placed position while the rater gradually increases the force into internal rotation until the lower leg “gives”.

The recorded degree of maximal force is conveyed to the assistant.

Protocol for rating of clinical hip osteoarthritis

Based on the findings from the clinical examination of passive hip range of motion and hip muscle strength, the rater assigns the hip to one of three categories: No hip OA, mild hip OA or severe hip OA.
